# Transcriptome and machine learning analysis of the impact of COVID-19 on mitochondria and multiorgan damage

**DOI:** 10.1371/journal.pone.0297664

**Published:** 2024-01-31

**Authors:** Yu-Yu Chang, An-Chi Wei

**Affiliations:** Graduate Institute of Biomedical Electronics and Bioinformatics, National Taiwan University, Taipei, Taiwan; University of Nebraska-Lincoln, UNITED STATES

## Abstract

The effects of coronavirus disease 2019 (COVID-19) primarily concern the respiratory tract and lungs; however, studies have shown that all organs are susceptible to infection by severe acute respiratory syndrome coronavirus 2 (SARS-CoV-2). COVID-19 may involve multiorgan damage from direct viral invasion through angiotensin-converting enzyme 2 (ACE2), through inflammatory cytokine storms, or through other secondary pathways. This study involved the analysis of publicly accessible transcriptome data from the Gene Expression Omnibus (GEO) database for identifying significant differentially expressed genes related to COVID-19 and an investigation relating to the pathways associated with mitochondrial, cardiac, hepatic, and renal toxicity in COVID-19. Significant differentially expressed genes were identified and ranked by statistical approaches, and the genes derived by biological meaning were ranked by feature importance; both were utilized as machine learning features for verification. Sample set selection for machine learning was based on the performance, sample size, imbalanced data state, and overfitting assessment. Machine learning served as a verification tool by facilitating the testing of biological hypotheses by incorporating gene list adjustment. A subsequent in-depth study for gene and pathway network analysis was conducted to explore whether COVID-19 is associated with cardiac, hepatic, and renal impairments via mitochondrial infection. The analysis showed that potential cardiac, hepatic, and renal impairments in COVID-19 are associated with ACE2, inflammatory cytokine storms, and mitochondrial pathways, suggesting potential medical interventions for COVID-19-induced multiorgan damage.

## Introduction

Patients with coronavirus disease 2019 (COVID-19) experience various respiratory issues. Acute respiratory distress syndrome (ARDS) due to COVID-19 pneumonia is the primary cause of mortality and long-term lung damage. Although the respiratory system is most commonly affected in people infected with severe acute respiratory syndrome coronavirus 2 (SARS-CoV-2), the virus can impact any organ in the body [1–3]. Indeed, multiple organs are typically involved in critically ill patients [4]. In addition to classical symptoms of respiratory distress, many patients with COVID-19 have systemic symptoms, including cardiovascular, hepatic or renal failure as well as coagulation disorders. Studies have reported organ damage involving the lungs (33% of patients), heart (32%), kidneys (12%), liver (10%), pancreas (17%), and spleen (6%); 66% of study participants had single- or multiorgan system damage, and 25% of patients showed multiorgan damage with varying degrees of overlap between various organs [5].

Cardiac complications occur in 20–44% of inpatients and are an independent risk factor for COVID-19-related death [6]. Viral invasion of cardiomyocytes [7] or systemic inflammatory responses without direct viral infiltration [8] can cause myocarditis, which can lead to heart failure and arrhythmias. Some patients with severe COVID-19, including those who did not have underlying kidney problems prior to the disease, acquire signs of kidney damage, with more than 30% of COVID-19 inpatients developing kidney damage [9]. In addition, multiple studies have reported the occurrence of liver damage in COVID-19 patients, indicating that 2–11% of COVID-19 patients develop liver comorbidities. Furthermore, in 16–53% of reported cases, increases in alanine aminotransferase (ALT) and aspartate aminotransferase (AST) levels occur during disease progression [10], which suggests that hepatocytes are damaged and that the liver is inflamed.

An inflammatory cytokine storm is the most frequently reported phenomenon in COVID-19. Inflammatory cytokines are immune responses intended to kill pathogens; however, the hyperinflammatory state associated with excessive production of cytokines can cause permanent damage to cells and mitochondria and induce cell death, potentially leading to further organ damage [11]. Angiotensin-converting enzyme 2 (ACE2), a key enzyme of the renin-angiotensin-aldosterone system (RAAS) that maintains homeostasis of blood pressure, electrolytes, and the inflammatory response, is also a possible cause of COVID-19-related damage to the lung [12], heart [13], liver [14] and kidney [15] as SARS-CoV-2 enters cells through ACE2. Mitochondria are another important target of SARS-CoV-2. Mitochondria, the main production sites of adenosine triphosphate (ATP) [16], are involved in the regulation of cellular immunity, homeostasis, and cell survival and death. There is evidence suggesting that SARS-CoV-2 hijacks the mitochondria of immune cells, replicates within the mitochondrial structure, and impairs mitochondrial dynamics, leading to cell death [17]. However, whether SARS-CoV-2 can impair organ function by direct viral infection via ACE2, mitochondrial damage, or multiorgan damage triggered by an inflammatory cytokine storm needs to be further investigated.

SARS-CoV-2 causes an increase in mitochondrial DNA (mtDNA) levels during infection that may trigger an excessive immune response and lead to severe pathology in COVID-19, including multiorgan failure [18]. While it is believed that mitochondrial antiviral signaling (MAVS) interacts with different SARS-CoV-2 proteins, the SARS-CoV-2 M protein inhibits MAVS protein aggregation, and the mitochondrial membrane-anchored MAVS protein is a key factor in the cellular antiviral defense system that further inhibits the innate antiviral response [19]. Immune evasion and hyperinflammation during COVID-19 can also be related to the disruption of mitochondrial quality [20]. In addition, patients with COVID-19 have reduced mitochondrial oxidative phosphorylation (OXPHOS) and bioenergetics, and COVID-19 is reportedly associated with inhibition of mitochondrial gene transcription [21]. SARS-CoV-2 infection hinders mitochondrial bioenergetics, which in turn can trigger inflammasome activation. Consequently, mitochondrial inhibition not only results in excessive cytokine production but also exerts a substantial impact on organs that heavily depend on mitochondrial energy production [21].

**Fig 1:**
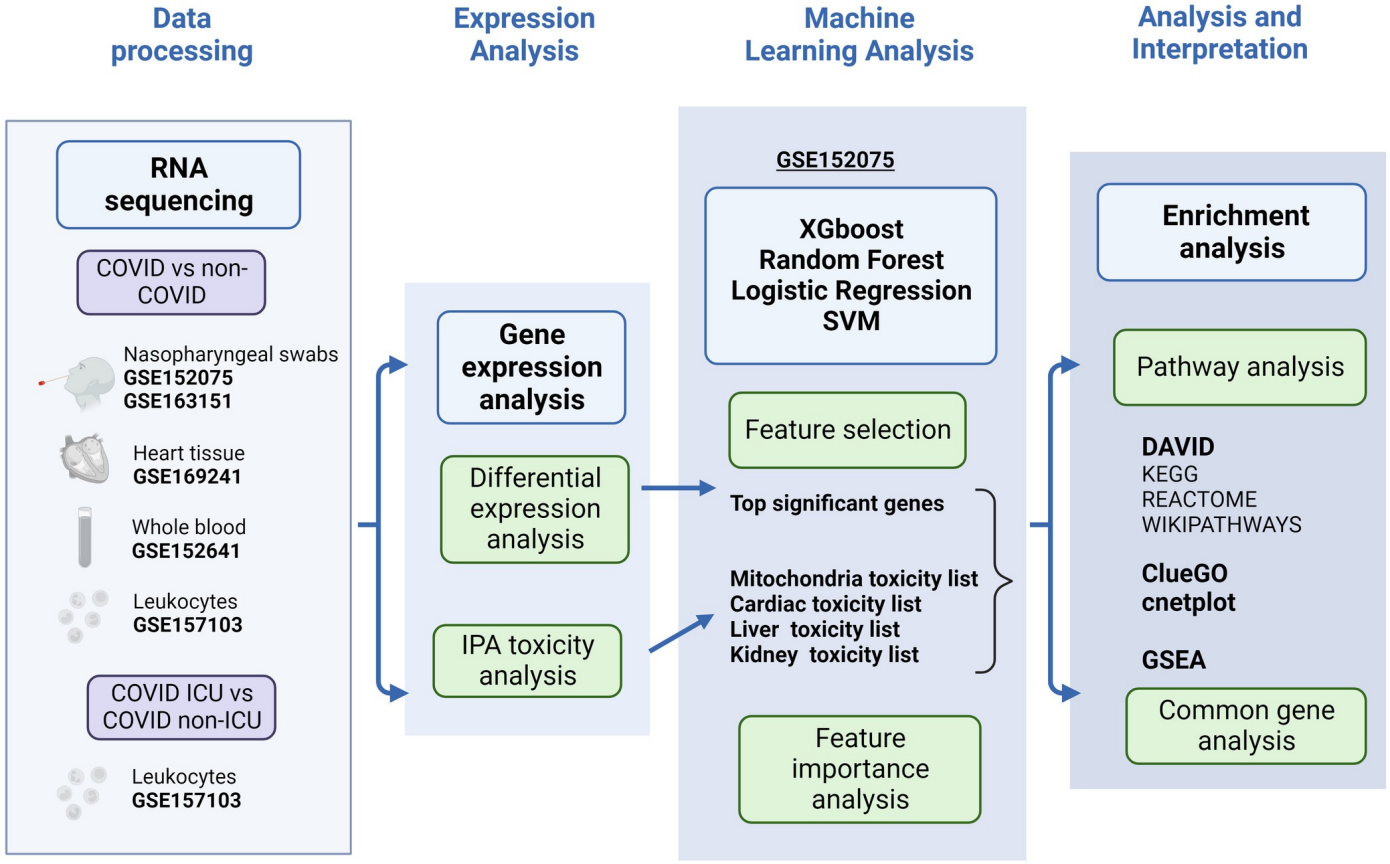
Summary flow chart. The analysis in this study starts with data collection on gene expression, followed by gene transcriptome analysis to obtain significantly expressed genes and toxicity analysis to further identify relevant significantly expressed genes. Subsequently, machine learning was used with the significantly expressed genes identified through statistical and biological methods to validate the hypothesis that SARS-CoV-2 would cause further damage to cardiac, hepatic, and renal function by infecting mitochondria. Finally, we conducted a literature review on the common genes, feature importance, and pathway analysis of these transcriptomes to investigate how SARS-CoV-2 infects mitochondria and damages cardiac, liver, and kidney function and drew conclusions. Created with BioRender.com.

Based on this evidence, we hypothesized that COVID-19 may cause damage to the heart, kidney, and liver via mitochondrial dysfunction and downstream responses in addition to direct viral infection and cytokine storms. Systemic transcriptomic analysis was conducted to examine the roles of mitochondria in COVID-19-related multiorgan damage (Fig 1). Our analysis shows that it can be reasonably inferred that there is a correlation between the mitochondrial damage caused by SARS-CoV-2 infection and further deterioration of heart, kidney, and liver function, resulting in multiorgan damage. This study enables understanding of the causes of multiorgan complications caused by SARS-CoV-2 and the development of treatment regimens.

## Materials and methods

Bioinformatics and machine learning tools were used to analyze multiple publicly available RNA-Seq sample sets from clinical samples. NetworkAnalyst is a comprehensive gene expression profiling and web visualization analysis [22]; Ingenuity Pathway Analysis (IPA) provides analysis and development tools for genomics, proteomics, drug toxicology, and metabolic and regulatory pathway studies [23]; DAVID (Database for Annotation, Visualization and Integrated Discovery) is a web-based tool for functional evaluation of the gene expression data [24–26]; ClueGO [27] is a Cytoscape plug-in for deciphering functionally grouped gene ontology and pathway annotation networks [28]; GSEA (Gene Set Enrichment Analysis) is applied to assess the distribution trend of genes in a specific gene set arranged in a gene table based on their correlation to the phenotype to determine its contribution to the phenotype [29, 30]. Python packages for machine learning sklearn (1.2.2), imblearn (0.10.1), and XGBoost (1.7.5) were run in Python 3.10.11. The cnetplot is a function from the clusterProfiler R package, commonly used for visualizing gene set enrichment analysis results. This function creates a category net plot that combines a category enrichment plot with a gene network plot. The version of clusterProfiler is 4.8.3 and runs in R 4.3.1; Venn diagrams and feature importance analysis tools were used to analyze specific genes to identify biological pathways and important molecules in the heart, kidney, and liver that correlate with mitochondria affected by SARS-CoV-2.

### Data availability

The raw counts of the RNA-seq data were obtained from the NCBI Gene Expression Omnibus (GEO) database. The selection of sample sets is determined by several factors, including the sample size for machine learning, the kind of human tissue being studied, and the presence of sufficient differentially expressed genes to facilitate further analysis. Prior to selecting these five sample sets, we conducted tests on additional sample sets. Nevertheless, the majority of these datasets exhibit a limited number of differentially expressed genes, typically fewer than 100 or slightly exceeding this threshold. Hence, after the evaluation based on the criteria, the selected sample sets comprised GSE152075, GSE163151, GSE157103, GSE169241, and GSE152641 for significant differentially expressed genes and toxicity analysis.

For the selection of sample sets of machine learning, our criteria were machine learning performance, sample size, imbalanced data state, and overfitting assessment. We also performed a comparison of GSE152075, GSE163151, GSE157103, and GSE152641 for machine learning performance and a complete analysis.

The sample sets GSE152075 and GSE163151 were obtained from nasopharyngeal swabs, GSE157103 from leukocytes, GSE169241 from human heart autopsy tissues, and GSE152641 from whole blood (Table 1). These five sample sets were first tested for differences between tissues. We then performed machine learning on GSE152075 with a larger sample size to validate the effect of COVID-19 on mitochondria and to identify further effects on the heart, kidney, and liver.

**Table 1:** Summary of RNA sequence sample sets of COVID-19 in Gene Expression Omnibus.

GEO accession	GSE title	Tissue	Platform	Sample size
GSE152075	In vivo antiviral host transcriptional response to SARS-CoV-2 by viral load, sex, and age [31]	Nasopharyngeal swab	GPL18573 Illumina NextSeq 500 (*Homo sapiens*)	484 (COVID: control = 430: 54)
GSE152075 Summary	SARS-CoV-2 triggered an antiviral response driven by interferon and concurrently decreased the transcription of ribosomal proteins. Levels of B cells and neutrophils were higher in patients with a lower viral load. A decrease in the expression of chemokines CXCL9/10/11, cognate receptors CXCR3, CD8A, and granzyme B was observed in elderly individuals. B- and NK-cell-specific transcripts were reduced and NF-κB inhibitors increased in males.			
GSE163151	A diagnostic host response biosignature for COVID-19 from RNA profiling of nasal swabs and blood [32]	Nasopharyngeal swab	GPL24676 Illumina NovaSeq 6000 (*Homo sapiens*)	149 (COVID: control = 138: 11)
GSE163151 Summary	A two-step classifier based on machine learning that was run on an individual test set of NP swab samples was able to segregate COVID-19 and non-COVID-19 and infectious or noninfectious acute respiratory disease with 85.1%-86.5% accuracy. SARS-CoV-2 infection has unique biologic features and differences between NP swabs and whole blood that can be used in the differential diagnosis of COVID-19.			
GSE169241	hPSC-derived cells to model macrophage-mediated inflammation in COVID-19 Hearts [33]	Human heart autopsy tissues	GPL24676 Illumina NovaSeq 6000 (*Homo sapiens*)	8 (COVID: control = 3:5)
GSE169241 Summary	COVID-19 patients showed a significant increase in CCL2 expression and macrophage infiltration in cardiac tissue. After SARS-CoV-2 exposure, macrophages induced increased cardiomyocyte reactive oxygen species and apoptosis via secretion of IL-6 and TNF-α.			
GSE157103	Large-scale multiomic analysis of COVID-19 severity [34]	Leukocyte	GPL24676 Illumina NovaSeq 6000 (*Homo sapiens*)	126 (COVID: control = 100: 26; ICU: non-ICU = 50: 50)
GSE157103 Summary	Coagulation-related proteins, as well as cellular fibronectin (cFN), were significantly increased in COVID-19 patients. The abundance of prothrombin, which is cleaved to form thrombin during coagulation, was significantly reduced and correlated with severity, which may help to elucidate the hypercoagulable environment of SARS-CoV-2 infection.			
GSE152641	Transcriptomic similarities and differences in host response between SARS-CoV-2 and other viral infection [35]	Whole blood	GPL24676 Illumina NovaSeq 6000 (*Homo sapiens*)	86 (COVID: control = 62: 24)
GSE152641 Summary	Changes in gene expression in the peripheral blood of COVID-19 patients correlated highly with changes in response to other viral infections. However, two genes, ACO1 and ATL3, showed significantly different changes in expression. Some dynamic immune evasion and counter host responses are specific to COVID-19. For example, CD56bright NK cells, M2 macrophages, and total NK cells were increased in COVID-19.			

### Processing of RNA sequencing data

NetworkAnalyst is utilized for gene expression analysis, and this study required tuning of various parameters. To initiate gene expression analysis using NetworkAnalyst, the mean is employed for gene-level summarization. It simplifies and standardizes representation, reduces random noise in the data, and makes it more amenable to downstream analyses. Additionally, it helps mitigate issues with multiple testing and operates under the assumption that each transcript contributes equally to the gene’s activity.

Before performing differential expression analysis, filtering is employed to enhance statistical power by eliminating genes that do not exhibit a response. To obtain accurate and meaningful inferences from differential expression analysis data, it is imperative to employ appropriate normalization techniques.

Filtering aids in the elimination of information that is demonstrably inaccurate or unlikely to be instructive. To modify the quantity of genes excluded from subsequent analysis, it is necessary to set up the parameters for variance and abundance filters. The variance filter eliminates features with a variance percentile rank less than the threshold of those with consistent expression values across circumstances. In this instance, the variance filtering was configured to a threshold of 15, in accordance with the default configuration of NetworkAnalyst. Consequently, data in the lowest 15th percentile of expression will be removed. The parameter for eliminating features with counts below the set threshold is referred to as low abundance. In this study, the default value of 4 was employed in NetworkAnalyst.

To accurately detect transcriptional differences and to guarantee that the expression distributions of each sample are consistent throughout the whole experiment, normalization is essential.

Log2-counts per million (Log2-CPM) is a normalization method commonly used in RNA-seq data processing. Log transformation helps to compress the range of data, ensuring that genes with high expression variability do not disproportionately influence subsequent analyses.

The limma approach is commonly utilized in the context of differential expression analysis because of its use of linear models, which frequently leads to improved computational efficiency when compared to alternative methods for differential expression analysis [36]. The adjusted p value was set to 0.05 [37] and the log_2_-fold change to 1.5 [38] to ensure that the differentially expressed genes being identified were statistically significant and biologically meaningful with sufficient variance. This makes the results more biologically relevant and valuable for application. The volcano plots of the significantly expressed genes in GSE152075, GSE169241, GSE157103, GSE163151, and GSE152641 show genes with increased and decreased gene expression (S1 Fig in S1 File) [39].

### Gene set enrichment and pathway analysis

After obtaining the significantly expressed genes from NetworkAnalyst’s differential gene expression analysis, we used Ingenuity Pathway Analysis (IPA; version 84978992) for a comparative analysis of the five sample sets and obtained toxicity lists for each sample set from the Tox analysis.

DAVID, ClueGO, the cnetplot from the clusterProfiler package in R and GSEA were used for pathway enrichment analysis and functional annotation. The significantly expressed genes of these transcriptomes were analyzed in terms of KEGG, REACTOME, and WIKIPATHWAYS in DAVID. Genes are linked with enriched pathways with GO, KEGG, and REACTOME by cnetplot. GSEA assesses how predefined gene sets are distributed within a gene table sorted by their correlation with the phenotype, helping to determine their impact on the phenotype. The gene set database utilized in the analysis was h.all.v2023.1.Hs.symbols.gmt. A total of 1000 permutations were performed, with gene symbols being collapsed in the database. The permutation type employed was phenotypic, and the chip platform used was Human_Gene_Symbol_with_Remapping_MSigDB.v2023.1.Hs.chip. ClueGO was used for comprehensive pathway and biological analysis and functional annotation of GO terms and pathways. The databases of GO, KEGG, WIKIPATHWAYS, REACTOME Reactions, and REACTOME Pathways were included in the ClueGO setting panel. The parameters encompass a p value threshold established at 0.05, a GO hierarchy ranging from level 8 to 15, and a pathway selection criterion of 2 genes with 6% per pathway. Other parameters were set to default. In addition, a Venn diagram was used to identify common genes for analysis of the biological meaning in the mitochondria, heart, kidney, and liver through their transcriptomes.

### Machine learning

The GSE152075 sample set was selected for machine learning based on several considerations. In numerous machine learning tasks, it is generally observed that the performance of the model tends to improve as the number of samples increases. This can be attributed to the fact that the model is subjected to a larger volume of data, enabling it to acquire a broader range of features and patterns. A greater number of data typically leads to improved generalization capabilities of the model.

When performing model training with a limited amount of data, there is an increased likelihood of encountering the phenomenon known as overfitting [40]. Overfitting refers to a situation in which the model demonstrates excellent performance when evaluated on the training data but fails to generalize effectively when presented with fresh, previously unseen data. This implies that the model could exhibit excessive complexity and has inflexibly acquired the characteristics of the training data, resulting in poor performance when applied to novel data.

The utilization of diverse sample sets may yield disparate outcomes, introducing complexity to the analysis. The GSE169241 sample set derives from human heart autopsy tissues, GSE157103 from leukocytes, and GSE152641 from whole blood samples. Similar to GSE152075, GSE163151 utilized nasopharyngeal swabs as the source of samples. However, due to the severe imbalance of samples between the COVID and control groups (138:11) in GSE163151, even employing techniques such as SMOTE to address the issue of imbalanced data might not produce adequate outcomes.

We use several indexes, including F1-Score, MCC, and AUC, to evaluate the performance of models, each with its specific formula, significance, and application, particularly in scenarios with imbalanced data.

The metric of F1-Score is the harmonic mean of precision and recall, calculated as

$$\text{F1-Score}=2\times \frac{\text{Precision}\times \text{Recall}}{\text{Precision}+\text{Recall}}$$

A higher F1-Score, closer to 1, indicates better model performance, balancing the precision and recall, which is especially crucial in imbalanced datasets. An F1-Score near 0 indicates poor model performance.

MCC (Matthews Correlation Coefficient) is calculated using the formula:

$$MCC=\frac{TP\times TN-FP\times FN}{\sqrt{\left(TP+FP\right)\left(TP+FN\right)\left(TN+FP\right)\left(TN+FN\right)}}$$

Where TP, TN, FP, and FN represent true positives, true negatives, false positives, and false negatives, respectively. MCC values range from -1 to +1. MCC is particularly valuable in imbalanced datasets as it provides a balanced measure even when class distribution is skewed.

AUC (Area Under the Curve) refers to the area under the ROC (Receiver Operating Characteristic) curve, AUC ranges from 0 to 1. AUC is less sensitive to class imbalance, making it a robust measure for evaluating models on imbalanced datasets.

In imbalanced data scenarios, these metrics are crucial as they offer more comprehensive insights into a model’s performance than mere accuracy. While a high accuracy might be misleading in such cases, a high score in F1, MCC, and AUC indicates that the model effectively handles both minority and majority classes, providing a holistic assessment of its predictive capabilities across different class distributions.

The machine learning comparison was conducted on four sample sets, GSE152075, GSE163151, GSE157103, and GSE1526414, with the top 40 significantly expressed genes (S1 Table in S1 File), which is explained for the number of gene selection in the subsection of sensitivity analysis of machine learning with varying significant gene counts. The GSE169241 sample set was excluded from the comparison due to its small sample size. Based on the factors mentioned above, the overall performance of GSE152075 generally showed the most optimized selection regarding machine learning performance, sample size, imbalanced data state, and overfitting assessment (S2 Table in S1 File).

In addition, utilizing several sample sets can offer a more comprehensive outlook; however, performing an in-depth examination of a singular sample set facilitates a more intricate study and comprehension. Therefore, we mainly utilized the GSE152075 sample set, which consists of 484 samples driven by the objective of ensuring consistency and uniformity in the subsequent study.

With the selected genes from the GSE152075 sample set as features, machine learning methods were used to test whether the association between COVID-19 and effects in the mitochondria, heart, kidney, and liver could be predicted. Through these machine learning results, the pathways and biological meanings of the genes were further analyzed.

We employed four machine learning algorithms, including XGBoost [41] with parameters for colsample_bytree = 0.9, learning_rate = 0.1, max_depth = 10, n_estimators = 50, random forest [42] with parameters for max_depth = 10, min_samples_split = 5, n_estimators = 100, logistic regression [43] with parameters for C = 50, max_iter = 5000 and SVM [44] with parameters for kernel = ’rbf’, C = 100, gamma = 0.01, probability = True, to test and predict COVID-19. The K-fold cross-validation technique was utilized, with K-fold sets to 10.

Since the ratio of the experimental group to the control group in the GSE152075 sample set was 430:54, the control group size was insufficient, which created a problem of imbalanced data. Thus, we employed SMOTE (Synthetic Minority Oversampling Technique) to analyze the samples in the minority category and add new samples to the sample set. SMOTE is a method used to address imbalanced sample sets, especially for oversampling minority classes. In imbalanced sample sets, the number of samples from one class greatly outnumbers the other. This results in many machine learning models being biased toward the majority class as they try to maximize overall accuracy, potentially neglecting or misclassifying the minority class. SMOTE addresses the issue of class imbalance not by simply duplicating samples from the minority class but by generating new synthetic samples. For every sample in the minority class, it identifies k-nearest neighbors, all of which belong to the same minority class. A neighbor is then randomly chosen, and a synthetic sample is produced at a random point between this sample and its selected neighbor. This procedure is iteratively carried out until a desired sample count or proportion for the minority class is reached. The synthesis method of SMOTE can be described using the following mathematical formula:

Given a sample *x*_*i*_ from the minority class, a random selection is made from its k nearest neighbors, denoted as *x*_*zi*_. Next, a random number λ between 0 and 1 is chosen. The new synthetic sample *x*_*new*_ can be generated using the formula:

$$x_{\text{new}}=x_{i}+\lambda \times (x_{zi}-x_{i})$$

This method ensures that the new synthetic sample lies somewhere on the line segment between the original sample and its chosen neighbor. Each iteration might yield different results because λ is randomized.

SMOTE offers notable advantages in tackling data imbalance. Instead of duplicating minority class samples, it produces synthetic samples, enhancing the sample set’s diversity and reducing the risk of overfitting. Furthermore, by expanding the minority class data, SMOTE ensures that models can better grasp the nuances of this class, leading to improved prediction accuracy [45]. Using this method, the ratio of the experimental group to the control group was 1:1.

SHAP (SHapley Additive exPlanations, 0.41.0) was used to analyze the prediction interpretation of the contribution of each feature. We then calculated the Shapley value of each feature to measure the contribution of the feature to the prediction so that the contribution of each feature could be understood in detail [46].

The codes are available at https://github.com/ntumitolab/ML-RNA-Seq.

## Results

After analyzing and processing the RNA sequencing raw count data for sample sets GSE152075, GSE163151, GSE169241, GSE157103 and GSE152641, the significant genes of each sample set were obtained and tested in machine learning, pathway analysis and IPA-Tox analysis.

### Sensitivity analysis of machine learning with varying significant gene counts

The initial investigation involved assessing the predictive capabilities of the features identified by the statistical methodology for COVID-19 to gain insights into the potential utility of machine learning. Consequently, we ranked the significantly expressed genes from the differential gene expression analysis of GSE152075 from NetworkAnalyst by their adjusted p value. The top 100 significantly expressed genes from the differential expression analysis of GSE152075 (S3 Table in S1 File) were ordered by the adjusted p value and used for machine learning and pathway analysis. The machine learning prediction power for predicting COVID-19 was tested on the top 100, 80, 60, 50, 40, 30, and 20 significantly expressed genes, and the results showed that the accuracy, F1-Score, and AUC of the four machine learning algorithms except SVM were near or above 90% for the top 30–100 significantly expressed genes (Table 2). This implies 30 significantly expressed genes to be sufficient for good prediction power. However, the overall performances of 40 significantly expressed genes in these four machine learning algorithms were better than 30 significantly expressed genes. Therefore, we proceeded with 40 genes for a more comprehensive pathway enrichment exploration.

**Table 2:** Machine learning results of the top 100, 80, 60, 50, 40, 30, 20 significantly expressed genes, and randomly selected 40 genes in GSE152075.

Features		100 genes	80 genes	60 genes	50 genes	40 genes	30 genes	20 genes	Randomly selected 40 genes
Machine Learning									
XGBoost	Accuracy	0.963	0.963	0.965	0.963	0.969	0.961	0.950	0.851
	Sensitivity	0.979	0.984	0.984	0.979	0.984	0.977	0.967	0.921
	Specificity	0.833	0.796	0.815	0.833	0.852	0.833	0.815	0.296
	Precision	0.979	0.975	0.977	0.979	0.981	0.979	0.977	0.912
	F1-Score	0.979	0.979	0.980	0.979	0.983	0.978	0.972	0.917
	MCC	0.812	0.807	0.819	0.812	0.842	0.804	0.758	0.225
	AUC	0.973	0.972	0.978	0.974	0.972	0.970	0.943	0.657
Random Forest	Accuracy	0.965	0.969	0.969	0.965	0.969	0.965	0.952	0.849
	Sensitivity	0.988	0.991	0.991	0.988	0.991	0.988	0.984	0.919
	Specificity	0.778	0.796	0.796	0.778	0.796	0.778	0.704	0.296
	Precision	0.973	0.975	0.975	0.973	0.975	0.973	0.964	0.912
	F1-Score	0.980	0.983	0.983	0.980	0.983	0.980	0.974	0.915
	MCC	0.815	0.837	0.837	0.815	0.837	0.815	0.745	0.220
	AUC	0.975	0.970	0.963	0.964	0.961	0.964	0.956	0.646
Logistic Regression	Accuracy	0.938	0.924	0.913	0.909	0.899	0.897	0.798	0.698
	Sensitivity	0.944	0.935	0.916	0.909	0.902	0.898	0.781	0.726
	Specificity	0.889	0.833	0.889	0.907	0.870	0.889	0.926	0.481
	Precision	0.985	0.978	0.985	0.987	0.982	0.985	0.988	0.918
	F1-Score	0.964	0.956	0.949	0.947	0.941	0.939	0.873	0.810
	MCC	0.737	0.676	0.669	0.667	0.628	0.631	0.487	0.143
	AUC	0.976	0.974	0.973	0.974	0.976	0.962	0.943	0.691
SVM	Accuracy	0.880	0.851	0.829	0.820	0.800	0.725	0.643	0.446
	Sensitivity	0.867	0.847	0.821	0.807	0.786	0.700	0.607	0.426
	Specificity	0.981	0.889	0.889	0.926	0.907	0.926	0.926	0.611
	Precision	0.997	0.984	0.983	0.989	0.985	0.987	0.985	0.897
	F1-Score	0.928	0.910	0.895	0.889	0.875	0.819	0.751	0.577
	MCC	0.638	0.546	0.511	0.517	0.480	0.408	0.337	0.023
	AUC	0.975	0.969	0.968	0.967	0.962	0.957	0.962	0.654

A randomly selected 40 genes from all genes of GSE152075 as machine learning features were also tested as a baseline to validate the result of assessing the predictive capabilities of the features identified by the statistical methodology for COVID-19. The result showed that the machine learning predictive capabilities cannot be established without pre-process feature selection.

**Fig 2:**
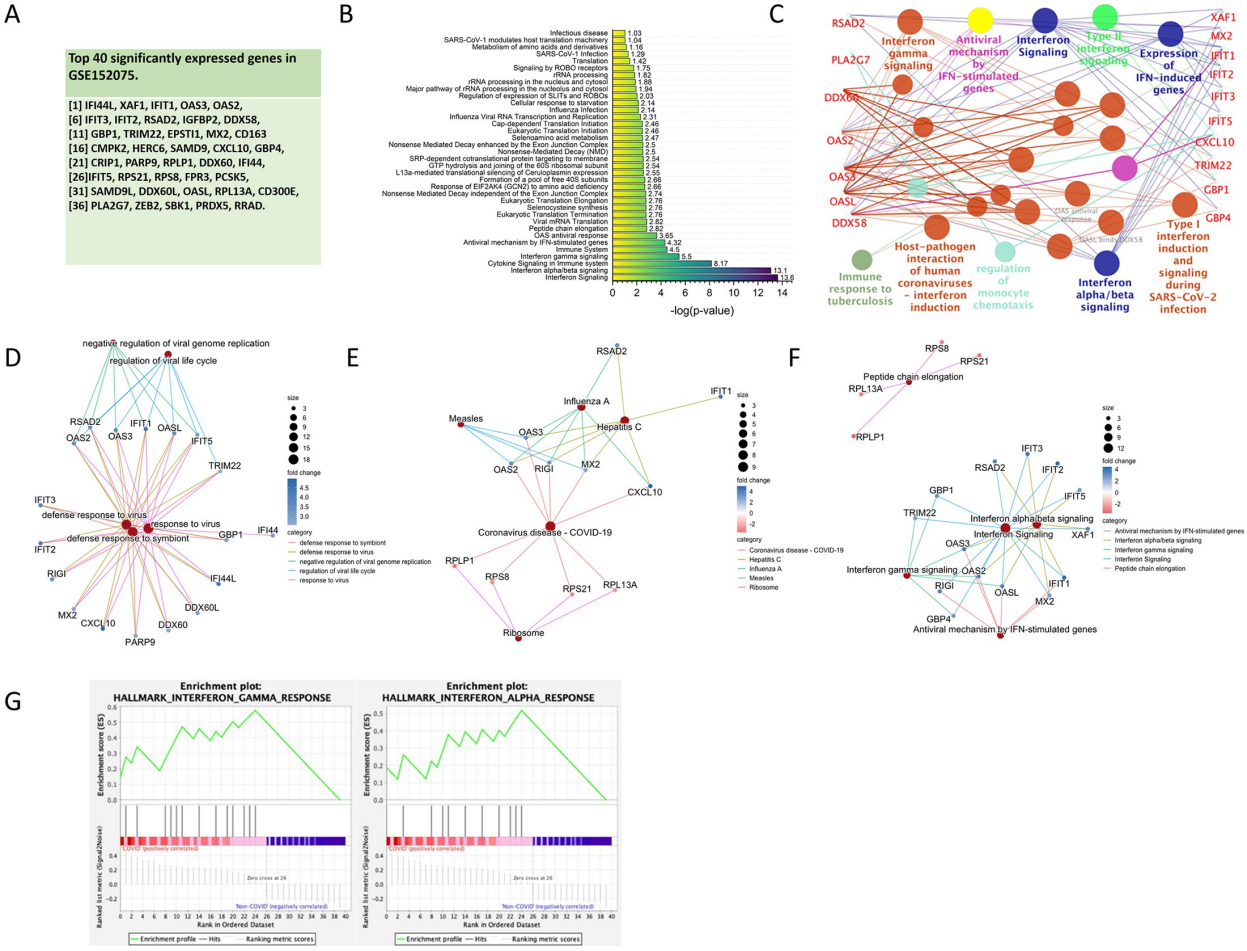
Pathway analysis of the top 40 significantly expressed genes in GSE152075. (A) List of the top 40 significantly expressed genes in the GSE152075 sample set. (B) DAVID analysis results of the top 40 significantly expressed genes in the REACTOME pathways. (C) Network of pathways from ClueGO for the top 40 significantly expressed genes showing that SARS-CoV-2 and inflammatory cytokine storm-related interferon signaling pathways are the main terms by group among the top 40 significantly expressed genes. Cnetplot of (D) GO enrichment analysis for the top 40 DEGs in Biological Process, (E) KEGG, and (F) REACTOME. (G) GSEA results for enrichment analysis of the top 40 significantly expressed genes.

The top 40 significantly expressed genes in the GSE152075 sample set (Fig 2A) were then analyzed in DAVID for KEGG, REACTOME, and WIKIPATHWAYS pathway analysis. COVID-19- and SARS-CoV-2-related pathways were identified as some of the major pathways from these top 40 significantly expressed genes. In addition, pathways related to inflammatory cytokine storms, such as interferon signaling [47], interferon alpha/beta/gamma signaling, and cytokine signaling in the immune system, were identified (Fig 2B), accounting for a large proportion of the pathway analysis results in ClueGo (Fig 2C). Genes linked with enriched pathways by cnetplot shows GO enrichment analysis for the top 40 DEGs in Biological Process (BP) is mainly virus-related pathway (Fig 2D), KEGG is coronavirus disease—COVID-19 (Fig 2E), and REACTOME is interferon-related signaling (Fig 2F). Interferon alpha/gamma signaling can also be seen in GSEA (Fig 2G). Hence, it can be inferred that inflammatory cytokine storms are strongly associated with COVID-19. Using these top 40 significantly expressed genes as machine learning features may effectively predict COVID-19.

### Tox analysis of mitochondria, heart, kidney, and liver

**Fig 3:**
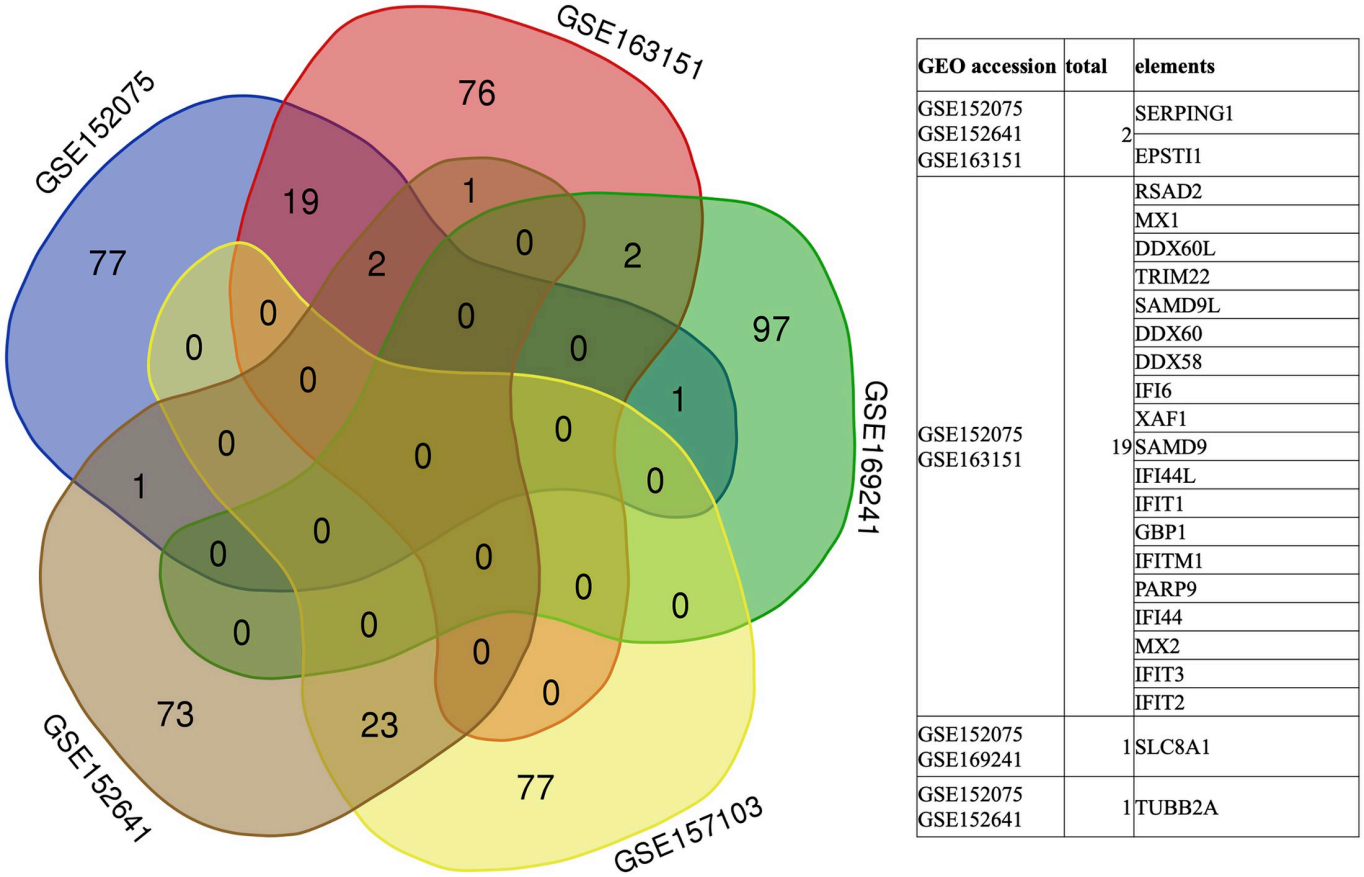
The common genes across the RNA-seq datasets. Top 100 significantly expressed genes were compared between RNA-seq datasets GSE152075, GSE163151, GSE169241, GSE157103 and GSE152641. The Venn diagram result indicates that the pathogenesis of COVID-19 is tissue specific.

The toxicity lists were obtained by performing Tox analysis on the significantly expressed genes in the five sample sets (Table 1) via IPA. The samples from different tissues and sampling platforms showed different common genes (Fig 3) and toxicity lists, indicating that the pathogenesis of COVID-19 is tissue specific.

**Fig 4:**
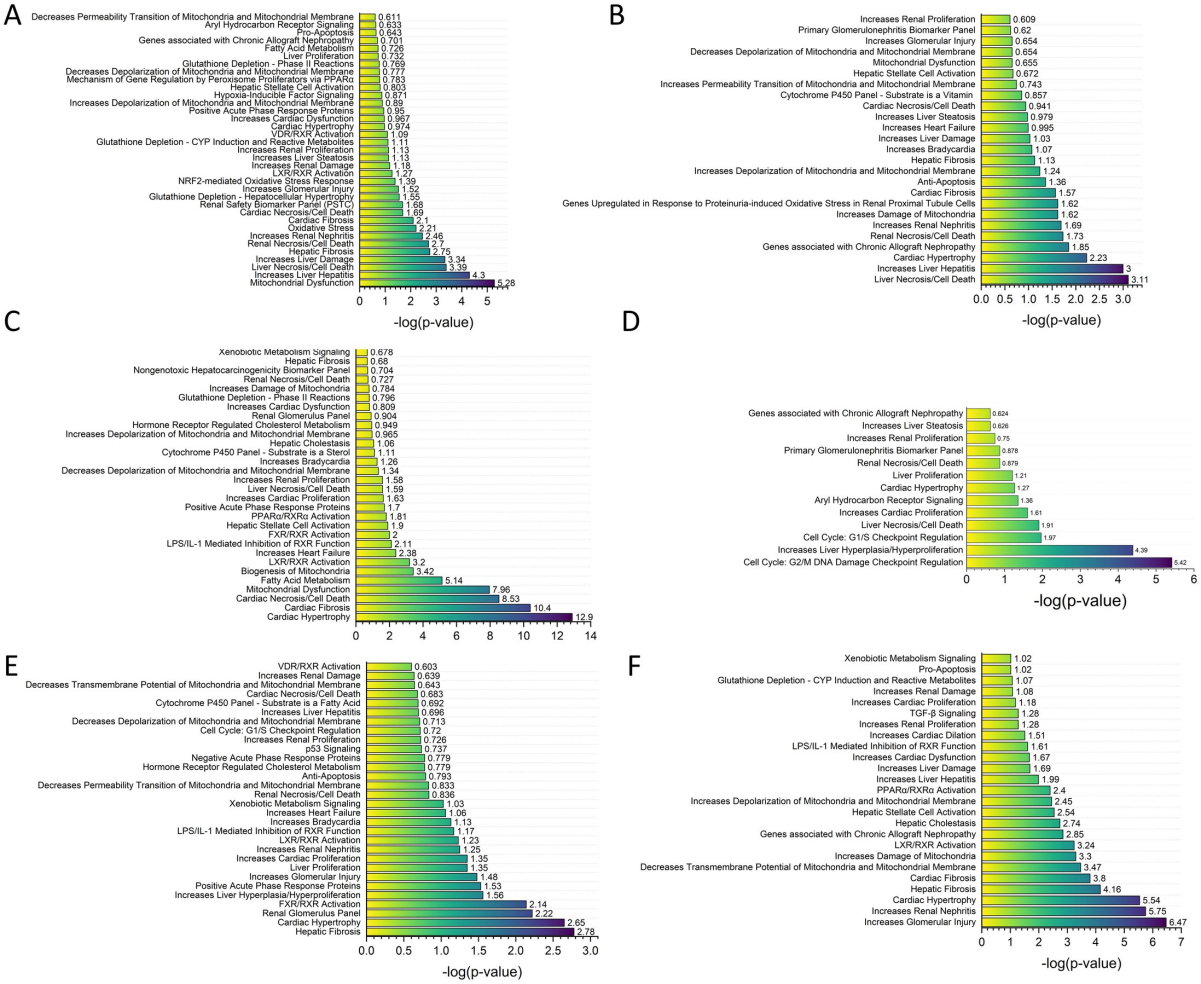
IPA comparative analysis of the RNA-seq data. Differentially expressed genes between the COVID-19 patients and the control groups were compared in the comparative IPA analysis for toxicity lists and toxicity functions. The following RNA-seq sample sets were obtained from the NCBI Gene Expression Omnibus database (GEO): GSE152075 (A, nasopharyngeal swab), GSE163151 (B, nasopharyngeal swab), GSE169241 (C, human heart autopsy tissues), GSE157103 (D, leukocyte), and GSE152641 (E, whole blood). ICU patients and non-ICU patients were compared in the GSE157103 sample set (F).

The nasopharyngeal swab data from GSE152075 and GSE163151 each showed the largest number of genes in common between these five sample sets and the toxic effects of COVID-19 on the heart, kidney, liver, and mitochondria (Fig 4A and 4B). The human heart tissue samples from GSE169241 showed the toxic effects of COVID-19 mainly on the heart and mitochondria (Fig 4C). While the data on leukocytes from GSE157103 and on whole-blood samples from GSE152641 showed that COVID-19 had toxic effects on the heart, kidney, and liver, the toxic effects on mitochondria were not significant (Fig 4D and 4E). However, the toxicity lists obtained from the GSE157103 leukocyte samples with ICU and non-ICU toxicity analysis showed a toxic mitochondrial effect in addition to the toxic cardiac, renal, and hepatic effects (Fig 4F), which suggests that mitochondrial dysfunction is associated with disease progression in COVID-19 patients [48].

### Machine learning of genes associated with the mitochondria-, heart-, kidney-, and liver-related toxicity list

In IPA-tox analysis of the GSE152075 sample set, we identified the differentially expressed genes (DEGs) associated with the mitochondria-related toxicity list (Table 3) under the criteria of -log(*p* value) > 1.3 for further machine learning analysis. Mitochondrial dysfunction was the relevant pathway identified by the mitochondria-related toxicity list, which included 32 DEGs in GSE150275. Similarly, significantly expressed genes were identified from the heart-, kidney-, and liver-related toxicity lists (Table 3). Among them, the heart-related toxicity lists included cardiac fibrosis [49] and cardiac necrosis/cell death pathways [50]; there were 38 genes in these toxicity lists. The kidney-related toxicity list included renal necrosis/cell death [51], increases renal nephritis [52] panel (PSTC) [53], and increases glomerular injury pathways [54]; there were 55 genes in these toxicity lists. The liver-related toxicity lists included increases in liver hepatitis [55], liver necrosis/cell death [56] and increases in liver damage pathways [57]; there were 42 genes in these toxicity lists (Table 3). These identified genes from the toxicity lists were compared to the top 100 and top 40 significantly expressed genes, and only a few common significantly expressed genes were identified, including *NDUFV1* and *PRDX5* in mitochondrial dysfunction and *RRAD*, *CIB1*, *CYBB*, and *SLC8A1* in the heart. There were many differences in gene expression between those identified by the statistical meaning method and those identified by the biological meaning method.

**Table 3:** Genes associated with mitochondria-, heart-, kidney-, and liver-related toxicity list and top significantly expressed genes in GSE152075.

	Genes associated with mitochondrial-related toxicity list (32 genes)	Genes associated with heart-related toxicity list (38 genes)	Genes associated with renal-related toxicity list (55 genes)	Genes associated with liver-related toxicity list (42 genes)
Related toxicity list	Mitochondrial Dysfunction	Cardiac Fibrosis Cardiac Necrosis/Cell Death	Renal Necrosis/Cell Death Increases Renal Nephritis Renal Safety Biomarker Panel (PSTC) Increases Glomerular Injury	Increases Liver Hepatitis Liver Necrosis/Cell Death Increases Liver Damage
Genes in toxicity list	ACO2, ATP5F1E, ATP5ME, BAD, CLIC2, COX4I1, COX5A, COX5B, COX6A1, COX7B, CYC1, FIS1, GPX1, GPX4, GSTP1, MT-ND6, NDUFA11, NDUFA13, NDUFA2, NDUFA4, NDUFAB1, NDUFB10, NDUFB2, NDUFB7, NDUFS6, NDUFV1, PRDX5, SURF1, TOMM7, UQCR11, UQCRC1, UQCRQ	CYBB, TLR4, PRKCB, SLC8A1, TLR2, GPX1, ACE2, DVL1, SLC2A1, STEAP3, CIB1, NDUFS6, MB, CARD6, JUN, LMNA, KLF15, LARP6, DAG1, USP18, NEXN, FLT1, PIN1, NUB1, CTNNB1, PROX1, S100A6, CCN1, RRAD, CASP1, BAD, NDUFA13, SOCS3, KLF4, NTN1, SERPINF1, JUND, ABCC9	MIF, TLR2, CLU, CTNNB1, FLT1, TLR7, TNFSF13B, TNFSF14, OLR1, LRP5, CYBB, GPX4, ZEB1, TLR4, P2RX7, KMO, ACE2, SLC2A1, FOS, LAMB2, PRKCB, MEFV, APRT, HSPA1A, HSPA1B, NDUFAB1, CASP1, STUB1, BAD, ALDH3B1, MLKL, BCL2L14, RFXANK, IDO1, SLC8A1, PRDX2, IER3, GNB2, BIRC3, CITED2, NTN1, GSTP1, ERBB2, ZBP1, APOBEC3A, AIM2, FCGR3A, FCGR3B, CX3CR1, TFF3, DDR1, CD274, JUN, C3AR1, CCR1	TLR4, GBP5, CASP1, CCL4, TLR2, TLR7, JUN, TNFSF14, ALDH3A1, P2RX7, KMO, MIF, BSG, CCR5, IL2RG, ATG4B, AIM2, CCN1, EPHA2, CCL2, KEAP1, CHCHD2, PROS1, SELL, KRT8, CTNNB1, FOS, CXCL10, IRF8, CD274, TKT, BAD, FGL2, GADD45B, SIGIRR, SOCS3, IER3, BIRC3, USP18, PTPRC, JUND, PHB2
Common genes with top 100 significantly expressed genes	NDUFV1, PRDX5	RRAD, CIB1, CYBB, SLC8A1	TNFSF13B, CCR1, CYBB, SLC8A1	ALDH3A1, CXCL10, GBP5
Common genes with top 40 significantly expressed genes	PRDX5	RRAD	_	CXCL10

Next, these DEGs in the mitochondria-, heart-, renal-, and liver-related toxicity lists were used as features to test the prediction efficiency of COVID-19 in machine learning models. The results showed that the gene sets in the mitochondria-, heart-, renal-, and liver-related toxicity list could mostly provide accuracy, F1-Score, and AUC of the four machine learning algorithms except SVM, which were near or above 90% (Table 4). These machine learning results demonstrated that the toxicity analyses of the mitochondria, heart, liver, and kidney correlated with COVID-19 and were sufficient for machine learning to predict COVID-19.

**Table 4:** Machine learning results of genes associated with the mitochondria-, heart-, kidney-, and liver-related toxicity list in GSE152075.

Feature		Genes selected from Mitochondrial dysfunction toxicity list (32 genes)	Genes selected from Heart toxicity list (Cardiac Fibrosis + Cardiac Necrosis/Cell Death) (38 genes)	Genes selected from Renal toxicity list (Renal Necrosis/Cell Death + Increases Renal Nephritis + Renal Safety Biomarker Panel (PSTC) + Increases Glomerular Injury) (55 genes)	Genes selected from Liver toxicity list (Increases Liver Hepatitis + Liver Necrosis/Cell Death + Increases Liver Damage) (42 genes)
Machine Learning					
XGBoost	Accuracy	0.948	0.938	0.938	0.942
	Sensitivity	0.981	0.970	0.974	0.967
	Specificity	0.685	0.685	0.648	0.741
	Precision	0.961	0.961	0.957	0.967
	F1-Score	0.971	0.965	0.965	0.967
	MCC	0.723	0.678	0.668	0.708
	AUC	0.859	0.913	0.913	0.933
Random Forest	Accuracy	0.944	0.952	0.940	0.944
	Sensitivity	0.991	0.993	0.986	0.981
	Specificity	0.574	0.630	0.574	0.648
	Precision	0.949	0.955	0.949	0.957
	F1-Score	0.969	0.974	0.967	0.969
	MCC	0.687	0.738	0.664	0.697
	AUC	0.922	0.955	0.960	0.969
Logistic Regression	Accuracy	0.905	0.897	0.907	0.909
	Sensitivity	0.930	0.907	0.912	0.914
	Specificity	0.704	0.815	0.870	0.870
	Precision	0.962	0.975	0.982	0.982
	F1-Score	0.946	0.940	0.946	0.947
	MCC	0.574	0.600	0.647	0.652
	AUC	0.863	0.898	0.919	0.939
SVM	Accuracy	0.936	0.899	0.771	0.723
	Sensitivity	0.979	0.919	0.756	0.693
	Specificity	0.593	0.741	0.889	0.963
	Precision	0.95	0.966	0.982	0.993
	F1-Score	0.964	0.942	0.854	0.816
	MCC	0.646	0.574	0.437	0.425
	AUC	0.935	0.949	0.937	0.966

To determine whether COVID-19 may further impair cardiac, hepatic, and renal function due to mitochondrial dysfunction, we added the 32 significantly expressed genes from the mitochondria-related toxicity lists to the significantly expressed genes of heart-, liver-, and kidney-related toxicity lists. After combining the lists, there were 66 significantly expressed genes related to the mitochondria and heart, 83 significantly expressed genes related to the mitochondria and kidney, and 73 significantly expressed genes related to the mitochondria and liver. The results of this round of machine learning indicated that the accuracy, F1 score, and AUC of all the machine learning algorithms, including SVM, using the three sets of transcriptomes were above 90% (Table 5). We found that adding the significantly expressed genes in the mitochondria-related toxicity list to those of the heart-, liver-, and kidney-related toxicity lists improved the prediction powers in machine learning models. In particular, the accuracy of the machine learning algorithm SVM of significantly expressed genes in the heart-, kidney-, and liver-related toxicity lists all improved notably (S2 Fig in S1 File). Thus, we can infer that COVID-19 may cause further damage to cardiac, liver, and kidney function by damaging mitochondria.

**Table 5:** Machine learning results of genes associated with mitochondria and other organ-related toxicity lists.

Features		Genes selected from Mitochondrial dysfunction + Heart (Cardiac Fibrosis + Cardiac Necrosis/Cell Death) toxicity list in GSE152075 (66 genes)	Genes selected from Mitochondrial dysfunction + Renal (Renal Necrosis/Cell Death + Increases Renal Nephritis + Renal Safety Biomarker Panel (PSTC) + Increases Glomerular Injury) toxicity list in GSE152075 (83 genes)	Genes selected from Mitochondrial dysfunction + Liver (Increases Liver Hepatitis + Liver Necrosis/Cell Death + Increases Liver Damage) toxicity list in GSE152075 (73 genes)
Machine Learning				
XGBoost	Accuracy	0.955	0.946	0.934
	Sensitivity	0.986	0.984	0.965
	Specificity	0.704	0.648	0.685
	Precision	0.964	0.957	0.961
	F1-Score	0.975	0.970	0.963
	MCC	0.755	0.707	0.661
	AUC	0.905	0.912	0.952
Random Forest	Accuracy	0.957	0.957	0.963
	Sensitivity	0.991	0.993	0.998
	Specificity	0.685	0.667	0.685
	Precision	0.962	0.960	0.962
	F1-Score	0.976	0.976	0.979
	MCC	0.764	0.763	0.799
	AUC	0.957	0.964	0.970
Logistic Regression	Accuracy	0.919	0.940	0.940
	Sensitivity	0.933	0.953	0.951
	Specificity	0.815	0.833	0.852
	Precision	0.976	0.979	0.981
	F1-Score	0.954	0.966	0.966
	MCC	0.657	0.727	0.732
	AUC	0.906	0.922	0.953
SVM	Accuracy	0.917	0.911	0.913
	Sensitivity	0.935	0.926	0.926
	Specificity	0.778	0.796	0.815
	Precision	0.971	0.973	0.975
	F1-Score	0.953	0.949	0.950
	MCC	0.638	0.628	0.641
	AUC	0.952	0.936	0.969

### Common gene analysis of the genes associated with mitochondria-, heart-, kidney-, and liver-related toxicity

**Fig 5:**
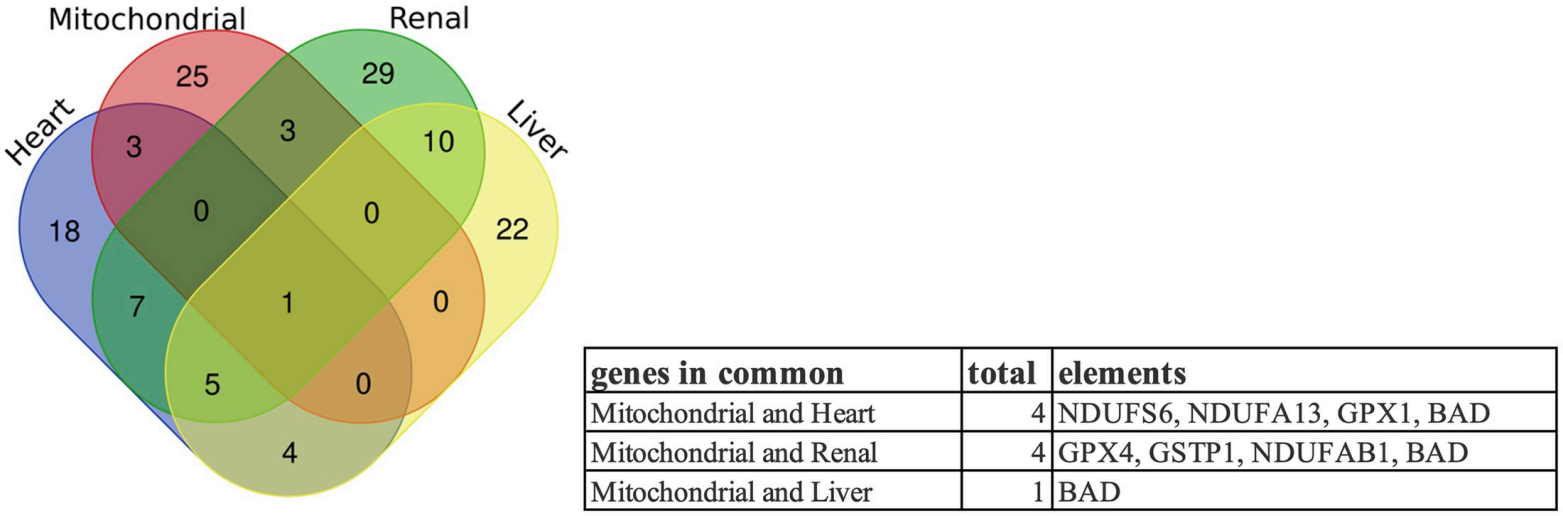
Common genes associated with mitochondria-, heart-, kidney-, and liver-related toxicity lists.

Finally, we identified the common genes among the significantly expressed genes from the mitochondria-related toxicity list and the three sets of significantly expressed genes from the heart-, kidney-, and liver-related toxicity lists for further analysis. The common genes in the mitochondria- and heart-related toxicity lists were *NDUFS6*, *NDUFA13*, *GPX1*, and *BAD*; the common genes in the mitochondria- and kidney-related toxicity lists were *GPX4*, *GSTP1*, *NDUFAB1*, and *BAD*. *BAD* (BCL2-associated agonist of cell death) was the only common gene between the mitochondria-related toxicity list and heart-, kidney-, and liver-related toxicity lists (Fig 5). The BAD protein, belonging to the Bcl-2 gene family, is a proapoptotic member involved in initiating apoptosis, which might explain the cell death in various tissue types and contribute to further pathogenicity and organ damage [58].

Regarding the common genes between the mitochondria-related toxicity lists and heart- and kidney-related toxicity lists, *GPX1* and *GPX4* were associated with oxidative stress; *NDUFAB1*, *NDUFS6*, and *NDUFA13* were associated with OXPHOS; and *GSTP1* was associated with the NRF2-mediated oxidative stress response. In addition to mitochondrial dysfunction, oxidative stress and the NRF2-mediated oxidative stress response were included in the GSE152075 toxicity lists. OXPHOS generates reactive oxygen species (ROS) [59]. The presence of excess ROS during the regulation of intracellular signaling may cause irreversible damage to cellular components and trigger apoptosis through the mitochondrial intrinsic apoptotic pathway [60]. Therefore, oxidative stress can cause apoptosis via a mitochondria-dependent pathway [61].

**Fig 6:**
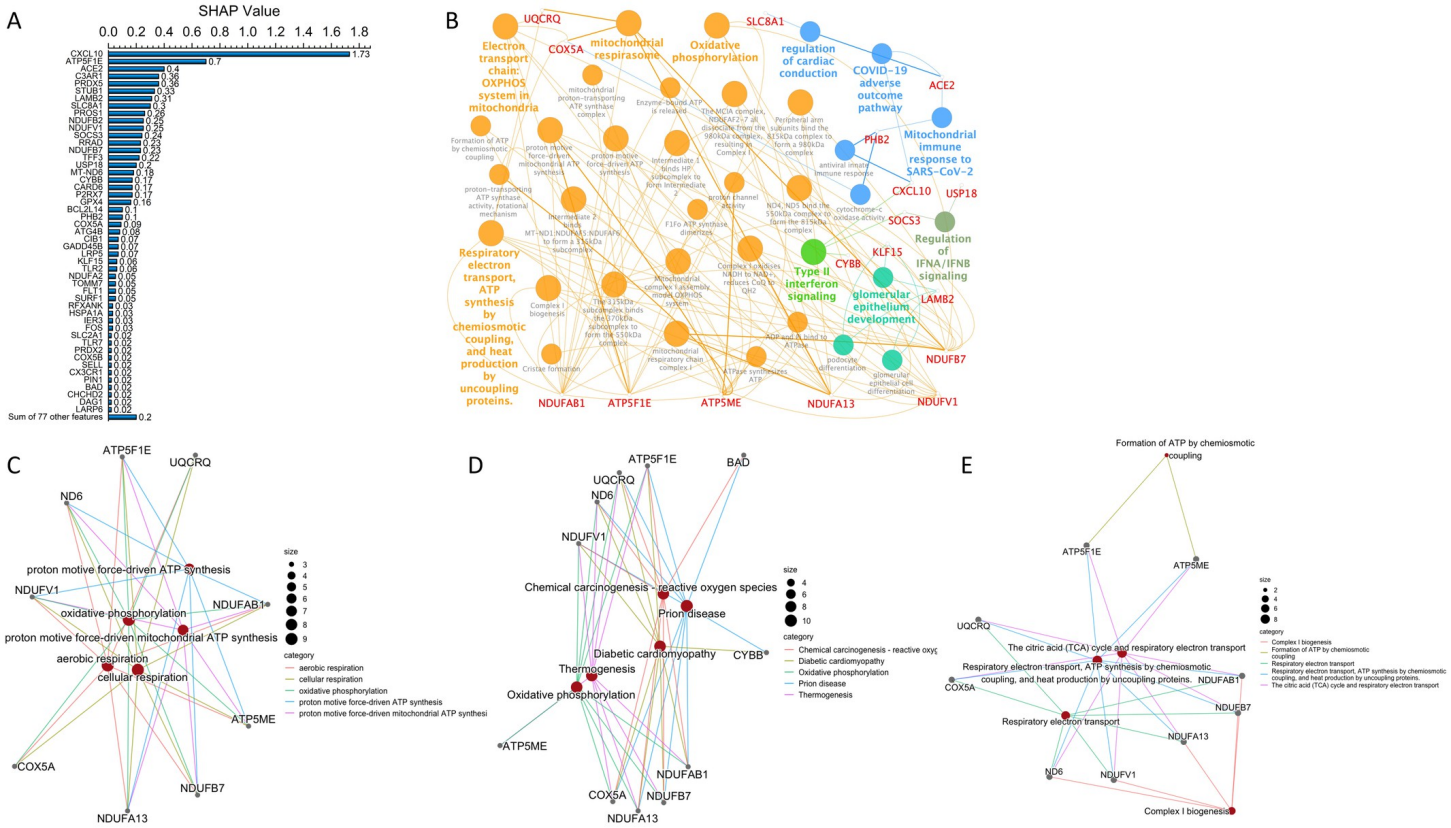
Feature importance analysis. (A) Feature importance is based on SHAP values of the genes from the mitochondria-, heart-, kidney-, and liver-related toxicity lists. (B) ClueGO network analysis of the top features with SHAP values higher than the average. SHAP was also used to identify feature importance genes in XGBoost to further elucidate the heart, kidney, and liver damage caused by mitochondrial infection triggered by COVID-19. Cnetplot of (C) GO enrichment analysis for the top features with SHAP values higher than the average in Biological Process, (D) KEGG, and (E) REACTOME.

SHAP was used to determine the feature importance of significantly expressed genes in the mitochondria-, heart-, kidney-, and liver-related toxicity lists to analyze which genes have the greatest impact. The top ranking feature importance of the significantly expressed genes *CXCL10*, *ATP5F1E*, and *ACE2* (Fig 6A) identified using the biological meaning method in the mitochondria-, heart-, kidney-, and liver-related toxicity lists. CXCL10 is associated with cytokine storms [62] and is an important chemokine [63]. Furthermore, it is reported to be an exceptional prognostic biomarker for COVID-19 patients [64, 65]. ACE2 is an entry receptor for SARS-CoV-2 and is also associated with mtDNA depletion and mitochondrial dysfunction [66]. ATP5F1E encodes a subunit of mitochondrial ATP synthase. A significant increase in expression of *ATP5F1E* in COVID-19 patients has been reported [67], which might be related to elevated production of ROS and increased inflammation [68, 69]. The network of pathways analysis from ClueGO involved the selection of the top feature importance of significantly expressed genes with SHAP values higher than the average. These genes include *CXCL10*, *RRAD*, *USP18*, *ATP5F1E*, *CIB1*, *C3AR1*, *CYBB*, *PROS1*, *ACE2*, *STUB1*, *UQCRQ*, *MT-ND6*, *SLC8A1*, *NDUFV1*, *COX5A*, *FLT1*, *NDUFA13*, *NDUFB7*, *BAD*, *ATP5ME*, *NDUFAB1*, *LAMB2*, *SOCS3*, *PHB2*, *TFF3*, *and KLF15*. The analyzed pathways show that the genes from mitochondria-, heart-, kidney-, and liver-related toxicity lists are closely related to COVID-19 and mitochondria, including the mitochondrial immune response to SARS-CoV-2, COVID-19 adverse outcome pathway, oxidative phosphorylation, type II interferon signaling, regulation of IFNA/IFNB signaling, and electron transport chain:OXPHOS system in mitochondria (Fig 6B). Genes linked with enriched pathways by cnetplot shows GO enrichment analysis for top feature importance of significantly expressed genes with SHAP values higher than the average in Biological Process (BP) is oxidative phosphorylation and ATP-related pathway (Fig 6C), KEGG is also oxidative phosphorylation (Fig 6D), and REACTOME is ATP-related pathway (Fig 6E). Thus, the top-ranking feature importance significantly expressed genes from mitochondria-, heart-, kidney-, and liver-related toxicity lists were identified by a biological meaning approach for machine learning analysis, and the main determinants included factors related to COVID-19, immune response and mitochondria. Several interferon signaling genes, such as *IFIT1*, *IFIT2*, *IFIT3*, *IFI5* and *CXCL10*, which are also associated with inflammatory cytokine storms and immune responses, were among the top 40 significantly expressed genes. Therefore, if the top significantly expressed genes were identified by a statistical meaning approach for machine learning analysis, the main determinants included factors also related to the inflammatory cytokine storm and immune response.

## Discussion

Machine learning is often applied for prediction or classification. Here, we employ machine learning in a reverse sense: we first formulated a hypothesis and then used the data that met the hypothesis to perform machine learning. If the accuracy of the prediction was high, the hypothesis had a high probability of being correct in terms of logical inference, which would provide an interpretation for the results obtained through machine learning.

The main objective of this study was to investigate the effects of COVID-19 on mitochondrial infection and subsequent damage to the heart, kidneys, and liver. To establish the correlation, two aims were hypothesized and then tested to determine the hypothetical validity. One aim used the statistical meaning approach and generated the prediction for machine learning, which would then be used as a baseline. This approach also allowed us to understand the reliability and rationality of machine learning when used on these sample sets. The other aim used the biological meaning of COVID-19 to analyze and elucidate the main hypothesis of our study, which regarded the infection of mitochondria by COVID-19 and subsequent cardiac, renal, and liver damage. Therefore, this study employed machine learning to evaluate our hypotheses.

The first aim used a statistical meaning approach to find relevant genes to be used as machine learning features. The machine learning results showed that by selecting the top significantly expressed genes for machine learning, it was possible to predict COVID-19 effectively, but this approach did not directly show the effect of COVID-19 on mitochondria, which might be because other factors have more direct effects. For example, inflammatory cytokine storms constituted a significant proportion in the DAVID analysis of the top 40 significantly expressed genes. Inflammatory cytokine storms have a strong connection with COVID-19, which causes multiorgan damage [70]. Machine learning can effectively predict COVID-19 using the top 40 significantly expressed genes as features.

The second aim tested the machine learning features selected from biological meaning, since the importance of individual genes in biological pathways and biological meaning do not entirely reflect the level of gene expression (i.e., few genes from the selected toxicity lists were shown in the top 100 significantly expressed genes). We used GSE152075 toxicity analysis to identify significantly expressed genes in the mitochondria-, heart-, kidney-, and liver-related toxicity lists. We then used machine learning to validate whether the significantly expressed genes identified by the biological meaning approach are able to predict COVID-19 and whether the infection of mitochondria by COVID-19 might have further biological meaning for potential cardiac, kidney, and liver damage. The results of the final analysis implied a correlation between the impact of COVID-19 on mitochondria and further cardiac, renal, and hepatic impairment. Therefore, we concluded that the effect of COVID-19 on mitochondria is associated with the potential impairment of cardiac, hepatic, and renal functions.

Machine learning has been widely applied to the diagnosis of COVID-19 patients. For example, the GSE152075 sample set has been used in other machine learning studies that used automated ML (AutoML) [71] and XGBoost for feature selection [72]. Among the 24 selected feature genes (*IGFBP2*, *KRT8*, *RPLP0*, *XAF1*, *RPL13*, *OAS2*, *CES1*, *RPL4*, *EEF1G*, *NR2F6*, *RPS8*, *RPL10A*, *SNX14*, *C5orf15*, *TNFRSF19*, *CD24*, *ALAS1*, *CEP112*, *C9orf24*, *POLR2J3*, *AAMP*, *DUOX2*, *EMCN*, and *RPL3*), keratin, type II cytoskeletal 8 (*KRT8*) was the only common gene out of the significantly expressed genes in the mitochondria-, heart-, kidney-, and liver-related toxicity lists obtained from our analysis of GSE152075 gene expression data. Maleknia et al. [73] employed the least absolute shrinkage and selection operator (LASSO) regression model to perform feature selection, and nasopharyngeal swab sample sets from GSE163151, GSE152075, GSE156063, and GSE188678 were applied. Random forest classification was used for training prediction. The common genes shared between those selected via feature selection using this LASSO regression model (*COPA*, *CXCL11*, *IFI6*, *MIF*, *NUCB1*, *SAMHD1*, *SIGLEC1*, and *TMED9*) and the top 100 significantly expressed genes of GSE152075 were interferon alpha inducible protein 6 (*IFI6*), C-X-C motif chemokine ligand 11 (*CXCL11*), and sialic acid binding Ig-like lectin 1 (*SIGLEC1*), all of which are related to the immune response. There was one gene in common when screening against all the significantly expressed genes in the GSE152075 mitochondria-, heart-, kidney-, and liver-related toxicity lists, which was macrophage migration inhibitory factor (MIF). Since different feature selection methods yield different genes, the biological pathways or biological meaning they represent can vary.

In our study, significantly expressed genes obtained from the statistical meaning approach and from the biological meaning approach had few genes in common. However, both machine learning results were predictive of COVID-19, and only the interpretation they presented differed [74]. As the features identified using the biological meaning approach were related to the mitochondria, heart, kidney, and liver, the interpretation of the machine learning results implied that the effects of SARS-CoV-2 on ACE2 and mitochondria were associated with further impairment of the heart, liver, and kidneys. One interpretation for the machine learning results using the statistical approach to feature selection was that SARS-CoV-2 triggers an inflammatory cytokine storm, which in turn impairs cardiac, hepatic, and renal function.

*BAD*, the only gene in common between the mitochondria-related toxicity list and the heart-, kidney-, and liver-related toxicity lists, is a regulator of apoptosis, and *BAD* mRNA expression is found in many tissues (heart, liver, spleen, lung, kidney, hypothalamus, pituitary, uterus, and ovary) [75]. Apoptosis occurs via the extrinsic death receptor pathway or the intrinsic intracellular pathway, which ultimately leads to mitochondrial dysfunction. In hepatocytes, the convergence of these cell death pathways also requires mitochondrial damages for effective apoptosis. The mitochondrial pathways of cell death are regulated by interactions among the Bcl-2 protein family members [76]. Liver biopsies from SARS patients have shown that SARS-CoV may induce apoptosis of hepatocytes, leading to liver damage [77]. Apoptosis leads to the deterioration of cardiac contractility observed in COVID-19 patients [78]. There is also evidence showing that COVID-19 causes renal tubular damage due to mitochondrial damage and apoptosis [79]. Therefore, mitochondrial dysfunction may be an important factor in apoptotic cell death that causes cardiac, kidney, and liver damage, and COVID-19 is an important cause of mitochondrial dysfunction.

Analysis of these common genes also showed that SARS-CoV-2 can hijack pathways, such as that of oxidative stress, after mitochondrial infection. Mitochondria are the main source of free radicals that are responsible for oxidative stress. If the antioxidant system fails to neutralize these free radicals in a timely manner, oxidative stress occurs, resulting in damage to cells and tissues. Mitochondria and mitochondrial DNA are targets of oxidative stress, as both the membrane structure and the inner components of mitochondria are susceptible to oxidative damage. When oxidative stress damages mitochondria, it affects cellular energy production and metabolism, which in turn affects all of the biological functions of cells and tissues. This stress causes apoptosis, which in turn is associated with impairment of cardiac, liver, and kidney function.

Our analysis revealed a correlation between the mitochondrial effects of COVID-19 and further impairment of cardiac, hepatic, and renal function. Mitochondrial dysfunction was determined to be a key factor in COVID-19 [80]. In addition, the analysis of gene expression in A549 and Calu3 cell lines infected with SARS-CoV-2 revealed an increase in the expression of genes related to cytokine production, inflammatory responses, mitochondria and autophagic processes [81]. Mitochondria cause cardiac dysfunction and myocyte damage via loss of metabolic capacity as well as via production and release of viral factors [82]. As our study focused on the analysis of cardiac, renal, and hepatic damage caused by the infection of mitochondria by SARS-CoV-2, we then used machine learning to validate whether the significantly expressed genes associated with mitochondria could be used to predict COVID-19 and to further analyze the damage to the heart, kidney, and liver caused by mitochondrial impairment.

Other studies have also shown that COVID-19 induces systemic host responses and transcriptomic changes and that the resulting disruptions affect the biological processes and functions of each organ system [83]. In addition, studies have shown that recovered patients show symptoms of long COVID with multiorgan damage [84–86]. Therefore, further investigation into whether the mitochondrial effects of COVID-19 also cause subsequent development of long COVID symptoms in patients is necessary [87].

## Conclusions

**Fig 7:**
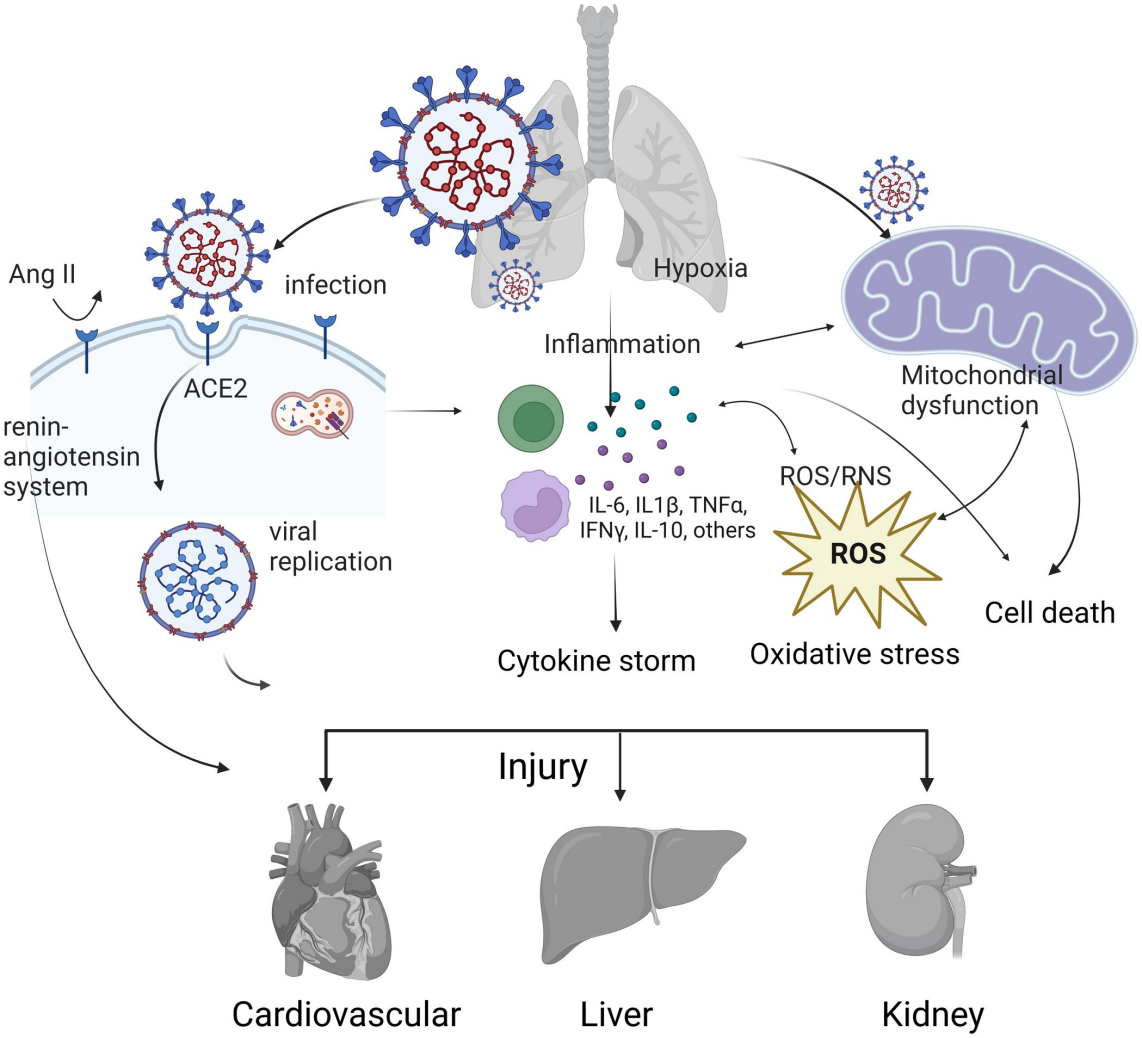
Mechanisms of COVID-19 causing multiorgan damage. SARS-CoV-2 can affect mitochondria, directly invade cells in various organs via ACE2, and lead to cytokine storms, oxidative stress, mitochondrial dysfunction and cell death, which can in turn impair cardiac, liver, and kidney function. The figure was created with BioRender.com.

An increasing number of case studies have recorded acute cardiac manifestations in patients with COVID-19. A significant proportion of patients diagnosed with COVID-19, with or without prior cardiovascular disease, demonstrate high levels of troponin or creatine kinase, suggesting myocardial injury, which in turn leads to cardiac insufficiency and arrhythmia [88]. The association between SARS-CoV-2 infection, mitochondrial dysfunction, and subsequent cardiovascular disease has been shown to be critical [89]. Similar findings have also been observed in other organs, such as the liver [90] and kidneys [91]. In this study, we demonstrated that SARS-CoV-2 can affect mitochondria, directly invade cells in various organs via ACE2, and trigger a cytokine storm, which in turn impairs cardiac, hepatic, and renal function (Fig 7). It is also possible that these correlations are because these pathways are related to each other. For example, direct infection by SARS-CoV-2 via ACE2-dependent pathways correlates with mitochondrial dysfunction [92], and there is a close association between mitochondrial dysfunction and immunosenescence; this may lead to an increased possibility of imbalance in the immune response to SARS-CoV-2 and may manifest as an exaggerated proinflammatory response and a cytokine storm [93], resulting in further multiorgan damage.

This study identified significantly expressed genes in the mitochondria-, heart-, kidney-, and renal-related toxicity lists from Tox analysis for machine learning to validate their association with COVID-19 and conclude that the mitochondrial infection caused by COVID-19 further impairs cardiac, hepatic, and renal function. We also obtained evidence for the correlation between genes, terms, and pathways in the mitochondria, heart, liver, and kidneys during COVID-19 that have been demonstrated in other studies. The aim of this study was to obtain the same conclusion using different methods that extended the inquiry and provided further interpretation for these findings. Although there are many possible mechanisms by which SARS-CoV-2 causes multiorgan damage, including direct cellular invasion via ACE2 and cytokine storms, which in turn impair cardiac, hepatic, and renal function, the hypothesis of this study, the correlation between the mitochondrial impact of COVID-19 and further cardiac, renal, and hepatic impairment, which was tested using machine learning, holds true.

By carrying out preliminary analysis through transcriptomics analysis, using machine learning to validate the conclusions of the analysis results, and cross-comparing reports, this analysis process identified and validated the hypotheses. However, importantly, the characteristics of the sample data used, such as the different tissues sampled and the different detection platforms and sample sizes, may affect the validation results. The trained machine learning models from GSE152075 with the top 40 significant genes were tested by other sample sets, including GSE163151, GSE157103, and GSE1526414. The result of prediction accuracy is notably poor, which implies that if the tissues, gene expression detection platforms, or parameter settings are different, the trained machine learning model cannot be utilized in other sample sets (S4 Table in S1 File). From this study, it can be seen that the results of toxicity lists varied between the different tissues sampled, and the results from the same tissues could also vary depending on the sampling platform and the ratio or size of the experimental and control samples. Further interpretation and the use of machine learning to analyze the effects of different sample tissues and different sample sizes on the hypothesis may be a valuable field of interest for subsequent study.

## Supporting information

S1 File (PDF)
