# Supplementary material for: Transcriptome and machine learning analysis of the impact of COVID-19 on mitochondria and multiorgan damage
Source: PLoS One. 2024 Jan 31;19(1):e0297664. doi: 10.1371/journal.pone.0297664 (PMC10830027; doi:10.1371/journal.pone.0297664)
Supplement: S1 File — (PDF) [file pone.0297664.s001.pdf]

Supporting information

S1 Table. Top 40 Significantly Expressed Genes in GSE152075, GSE163151, GSE157103, and GSE1526414.

Top 40 significantly expressed genes			
**GSE152075**	**GSE163151**	**GSE157103**	**GSE152641**
EPSTI1, LINC00324, IFI6, IFI44, FTH1P3, IFIT1, EIF2AK2, DDX60, IFITM1, IRAK1, SP100, RSAD2, SAMD9, LOC283214, DSC3, ATP2A2, MAP2K7, CHD1, BST2, RNF125, SNORD119, ESR1, IFI27, NT5C3A, LRRC8D, SNORA12, TTC30A, LETM1, CAPN12, MOV10L1, FBXO31, C15orf61, XAF1, YPEL1, MFGE8, SAMD9L, PARP14, MALAT1, TRUB2, AP3B1	EPSTI1, LINC00324, IFI6, IFI44, FTH1P3, IFIT1, EIF2AK2, DDX60, IFITM1, IRAK1, SP100, RSAD2, SAMD9, LOC283214, DSC3, ATP2A2, MAP2K7, CHD1, BST2, RNF125, SNORD119, ESR1, IFI27, NT5C3A, LRRC8D, SNORA12, TTC30A, LETM1, CAPN12, MOV10L1, FBXO31, C15orf61, XAF1, YPEL1, MFGE8, SAMD9L, PARP14, MALAT1, TRUB2, AP3B1	CDC6, PBK, DTL, DEPDC1B, MELK, MCM10, TOP2A, RRM2, GINS1, MCM6, BUB1, CLSPN, CDC45, ESCO2, CDC25A, ASPM, MKI67, STIL, TYMS, BUB1B, CDCA2, CCNA2, NCAPG, TRIP13, NUSAP1, CDK1, MCM4, KIF15, EXO1, ZWINT, CHEK1, KIF11, GTSE1, DSCC1, E2F7, HJURP, PLK4, GLDC, NDC80, ORC1	POLQ, CDC6, CLSPN, EPSTI1, CDCA2, RRM2, MCM10, H2BC6, IFI27, DHCR24, MS4A4A, H2AC4, DTL, TTC9, KIFC1, P2RY10, APOBEC3A, NT5E, CC2D2A, TIMM10, TOP1MT, GTSE1, SLC26A8, AMIGO1, GPR141, H2BC3, H3C3, INSC, CARD17, TWIST2, H2BC9, H3C8, TMEM144, TRIP13, AK4, FCER1A, HPD, METTL7B, H3C12, H3C7

S2 Table. Sample Set Selection for Machine Learning and Further Analysis. (5) Overall Ranking Score = (1) +(2) + (3) + (4).

Machine Learning \ Feature		Machine Learning for Top 40 sig genes of GSE152075	Machine Learning for Top 40 sig genes of GSE163151	Machine Learning for Top 40 sig genes of GSE157103	Machine Learning for Top 40 sig genes of GSE152641
XGBoost	Accuracy	0.969	0.973	0.897	0.884
	Sensitivity	0.984	0.986	0.920	0.919
	Specificity	0.852	0.818	0.808	0.792
	Precision	0.981	0.986	0.948	0.919
	F1-Score	0.983	0.986	0.934	0.919
	MCC	0.842	0.804	0.700	0.711
	AUC	0.972	0.991	0.886	0.958
	(1) Machine Learning Performance Ranking	(1)	(1)	(3)	(4)
	(2) Sample Size and Ranking	484 (1)	149 (2)	126 (3)	86 (4)
	(3) Imbalance Data Size and Ranking	(430: 54 => 11.16%) (3)	(138: 11 => 7.38%) (4)	(100: 26 => 20.63%) (2)	(62: 24 => 27.91%) (1)
	(4) Overfit Checking by Learning Curve	Pass (1)	Pass (1)	Might be Overfitting (2)	Might be Overfitting (2)
	(5) Overall Ranking Score and Ranking	(6) **1**	(8) **2**	(10) **3**	(11) **4**
Random	Accuracy	0.969	0.980	0.865	0.930

Forest	Sensitivity	0.991	1.000	0.890	0.952
	Specificity	0.796	0.727	0.769	0.875
	Precision	0.975	0.979	0.937	0.952
	F1-Score	0.983	0.989	0.913	0.952
	MCC	0.837	0.844	0.619	0.827
	AUC	0.961	0.917	0.939	0.995
	(1) Machine Learning Performance Ranking	(1)	(1)	(4)	(3)
	(2) Sample Size and Ranking	484 (1)	149 (2)	126 (3)	86 (4)
	(3) Imbalance Data Size and Ranking	(430: 54 => 11.16%) (3)	(138: 11 => 7.38%) (4)	(100: 26 => 20.63%) (2)	(62: 24 => 27.91%) (1)
	(4) Overfit Checking by Learning Curve	Might be Overfitting (2)	Might be Overfitting (2)	Might be Overfitting (2)	Might be Overfitting (2)
	(5) Overall Ranking Score and Ranking	(7) **1**	(9) **2**	(11) **4**	(10) **3**
Logistic Regression	Accuracy	0.899	0.973	0.857	0.977
	Sensitivity	0.902	0.986	0.860	0.968
	Specificity	0.870	0.818	0.846	1.000
	Precision	0.982	0.986	0.956	1.000
	F1-Score	0.941	0.986	0.905	0.984
	MCC	0.628	0.804	0.633	0.945
	AUC	0.976	0.984	0.866	0.987
	(1) Machine Learning Performance Ranking	(2)	(1)	(4)	(3)
	(2) Sample Size and Ranking	484 (1)	149 (2)	126 (3)	86 (4)

	(3) Imbalance Data Size and Ranking	(430: 54 => 11.16%) (3)	(138: 11 => 7.38%) (4)	(100: 26 => 20.63%) (2)	(62: 24 => 27.91%) (1)
	(4) Overfit Checking by Learning Curve	Pass (1)	Pass (1)	Might be Overfitting (2)	Might be Overfitting (2)
	(5) Overall Ranking Score and Ranking	(7) **1**	(8) **2**	(11) **4**	(10) **3**
	Accuracy	0.800	0.973	0.865	0.953
	Sensitivity	0.786	0.993	0.860	0.952
	Specificity	0.907	0.727	0.885	0.958
	Precision	0.985	0.979	0.966	0.983
	F1-Score	0.875	0.986	0.910	0.967
	MCC	0.480	0.790	0.662	0.889
	AUC	0.962	0.914	0.949	0.987
SVM	(1) Machine Learning Performance Ranking	(2)	(1)	(4)	(3)
	(2) Sample Size and Ranking	484 (1)	149 (2)	126 (3)	86 (4)
	(3) Imbalance Data Size and Ranking	(430: 54 => 11.16%) (3)	(138: 11 => 7.38%) (4)	(100: 26 => 20.63%) (2)	(62: 24 => 27.91%) (1)
	(4) Overfit Checking by Learning Curve	Pass (1)	Might be Overfitting (2)	Might be Overfitting (2)	Might be Overfitting (2)
	(5) Overall Ranking Score and Ranking	(7) **1**	(9) **2**	(11) **4**	(10) **3**
The Sample Set Selection for Further Analysis		**X**			

S3 Table. Top 100 Significantly Expressed Genes in GSE152075.

Top 100 significantly expressed genes in GSE152075.
[1] IFI44L, XAF1, IFIT1, OAS3, OAS2, IFIT3, IFIT2, RSAD2, IGFBP2, DDX58,<br><br>[11] GBP1, TRIM22, EPSTI1, MX2, CD163, CMPK2, HERC6, SAMD9, CXCL10, GBP4,<br><br>[21] CRIP1, PARP9, RPLP1, DDX60, IFI44, IFIT5, RPS21, RPS8, FPR3, PCSK5,<br><br>[31] SAMD9L, DDX60L, OASL, RPL13A, CD300E, PLA2G7, ZEB2, SBK1, PRDX5, RRAD,<br><br>[41] OAZ1, SLAMF7, RPS5, WARS1, ANAPC11, CXCL9, MX1, TNFSF13B, DTX3L, CKB,<br><br>[51] FAU, CYBB, RPLP2, C9orf24, ATP5IF1, RPL13, SIGLEC1, MS4A7, H2AJ, HERC5,<br><br>[61] TCTEX1D2, TRIM5, RPLP0, OAS1, RPL18A, MS4A6A, RPS28, SPINT2, CIB1, TUBB2A,<br><br>[71] ZMYND10, CXCL11, NDUFV1, SLC8A1, SERPING1, RPS19, CD68, UBE2L6, CAPS, PTPRN2,<br><br>[81] FAM166B, GBP5, RPL10A, IFITM1, SSR4, SLC25A6, SMIM22, MARCHF1, RPL3, ALDH3A1,<br><br>[91] CLDN7, RPL35, KLRD1, CCR1, IFI6, MSR1, EEF2, RPS3A, GUK1, LAMTOR4

S4 Table. The Prediction Accuracy of Learned ML Models from GSE152075 with the Top 40 Significant Genes Tested in GSE152075, GSE163151, GSE157103, and GSE1526414.

Features / Prediction accuracy of learned ML models from GSE152075	Top 40 significant genes of GSE152075 in GSE163151	Top 40 significant genes of GSE152075 in GSE157103	Top 40 significant genes of GSE152075 in GSE152641
XGBoost	0.315	0.754	0.721
Random Forest	0.215	0.619	0.581
Logistic Regression	0.765	0.556	0.453
SVM	0.832	0.563	0.383

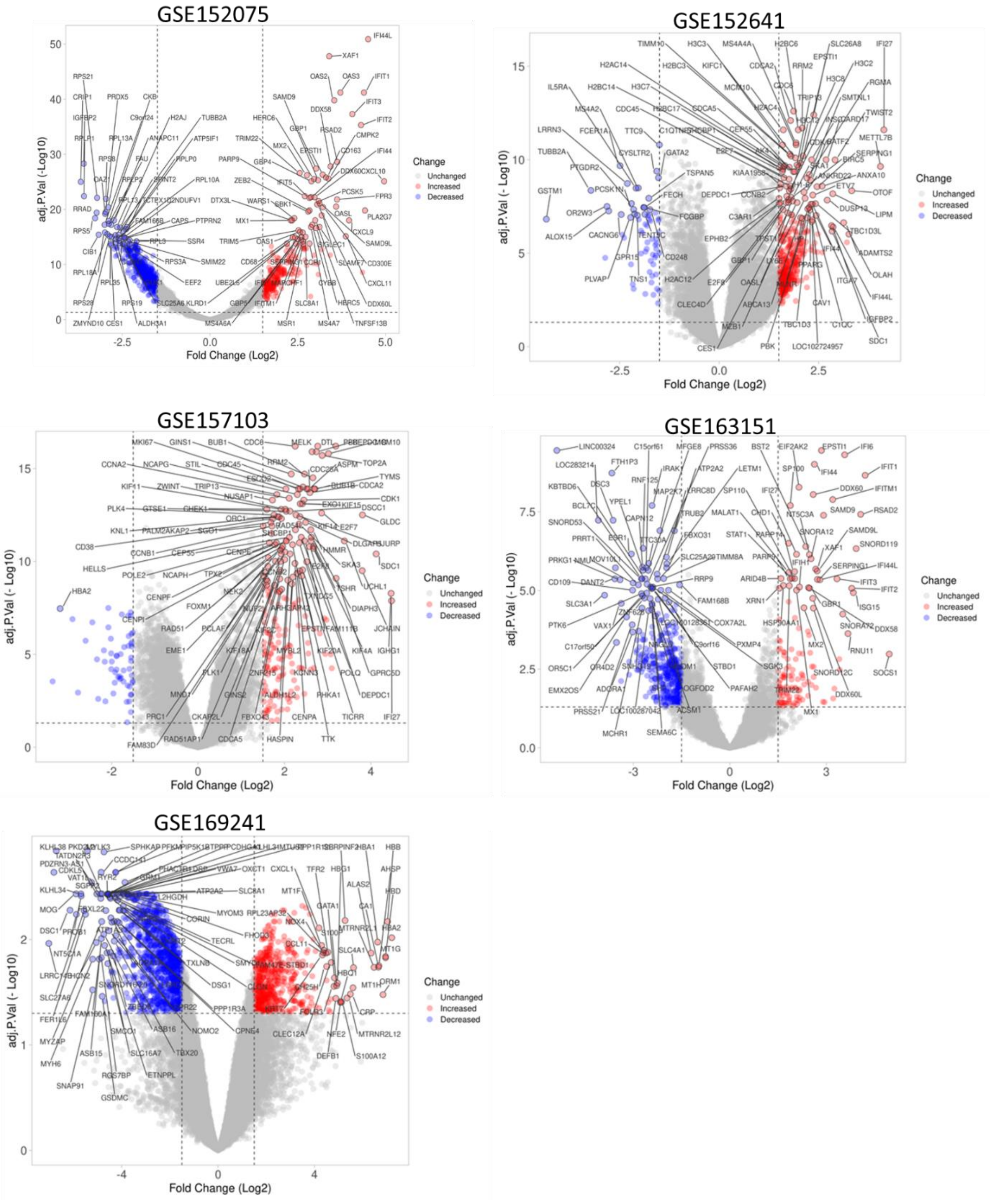

S1 Fig. Volcano Plots of the Significantly Expressed Genes in GSE152075, GSE169241, GSE157103, GSE163151, and GSE152641 by VolcaNoseR.

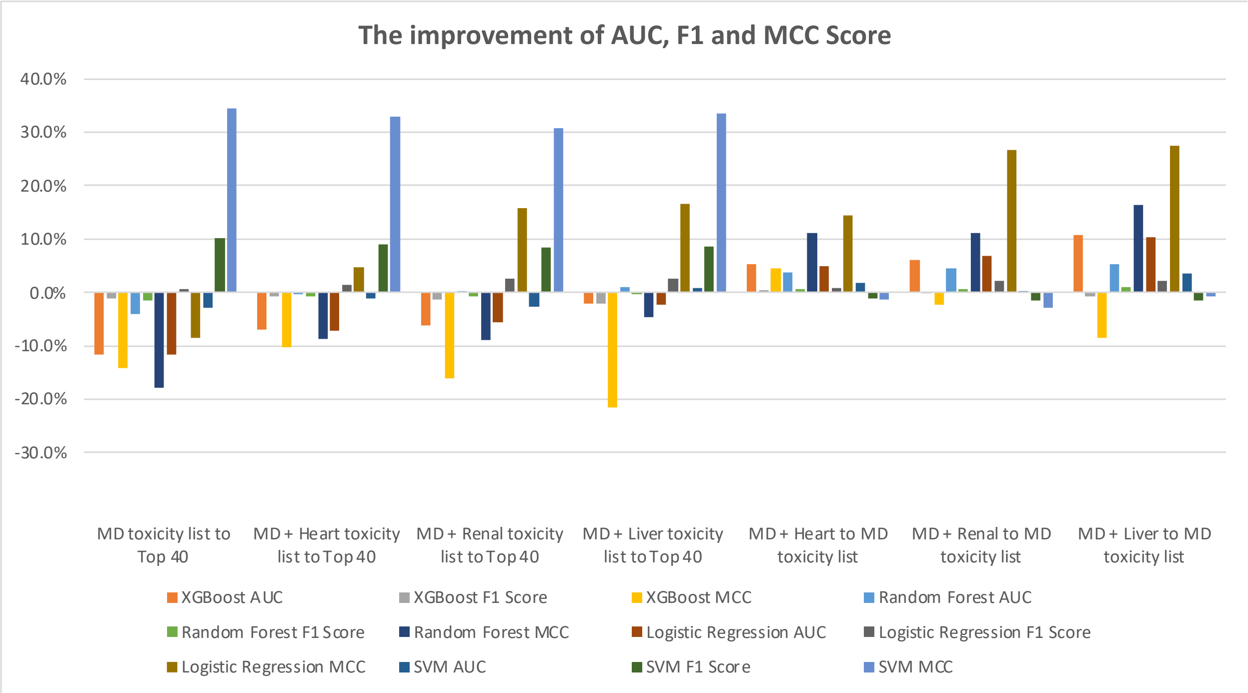

S2 Fig. The Improvement of Adding Heart-, Kidney-, and Liver-Related Toxicity Genes in Mitochondrial Dysfunction and the Comparison between the Top 40 Significant Genes of GSE152075 and Mitochondrial Dysfunction. MD represents mitochondrial dysfunction, and the top 40 represent the top 40 significant genes of GSE152075 in the table.
